# Acute stress in residents playing different roles during emergency simulations: a preliminary study

**DOI:** 10.5116/ijme.5929.60f1

**Published:** 2017-06-19

**Authors:** Roger Daglius Dias, Augusto Scalabrini-Neto

**Affiliations:** 1 Emergency Department, Hospital das Clínicas, University of São Paulo Medical School, São Paulo, Brazil

**Keywords:** Simulation, acute stress, multidisciplinary training, role-play

## Abstract

**Objectives:**

To investigate acute stress response in residents playing nurse and physician roles during emergency simulations.

**Methods:**

Sixteen second-year internal medicine residents participated in teams of four (two playing physician roles and two playing nurse roles). Stress markers were assessed in 24 simulations at baseline (T1) and immediately after the scenario (T2), using heart rate, systolic and diastolic blood pressure, salivary α-amylase, salivary cortisol and salivary interleukin-1β. The State-Trait Anxiety Inventory was applied at T2. Continuous data were summarized for the median (1st-3rd interquartile ranges), and the Mann-Whitney U Test was used to compare the groups.

**Results:**

The percent variations of the stress markers in the physician and nurse roles, respectively, were the following: heart rate: 70.5% (46.0-136.5) versus 53.0% (29.5-117.0), U=89.00, p=0.35; systolic blood pressure: 3.0% (0.0-10.0) versus 2.0% (-2.0-9.0), U=59.50, p=0.46; diastolic blood pressure: 5.5% (0.0-13.5) versus 0.0% (0.0-11.5), U=91.50, p=0.27; α-amylase: -5.35% (-62.70-73.90) versus 42.3% (12.4-133.8), U=23.00, p=0.08; cortisol: 35.3% (22.2-83.5) versus 42.3% (12.4-133.8), U=64.00, p=0.08); and interleukin-1β: 54.4% (21.9-109.3) versus 112.55% (29.7-263.3), U= 24.00, p=0.277. For the physician and nurse roles, respectively, the average heart rate was 101.5 (92.0-104.0) versus 91.0 (83.0-99.5) beats per minute, U=96.50, p=0.160; and the state anxiety inventory score was 44.0 (40.0-50.0) versus 42.0 (37.50-48.0) points, U= 89.50, p=0.319.

**Conclusions:**

Different roles during emergency simulations evoked similar participants’ engagement, as indicated by acute stress levels. Role-play strategies can provide high psychological fidelity for simulation-based training, and these results reinforce the potential of role-play methodologies in medical education.

## Introduction

Healthcare simulation-based education has become an essential component in undergraduate, graduate and continuing medical training.^1^^,^^2^ High-fidelity simulation (HFS) is an indispensable tool in virtually every field of medicine.^3^^,^^4^ The primary benefit of using HFS is that learners can practice on multiple levels (cognitive, procedural and affective) in a safe environment, where errors will not harm real patients.^5^^,^^6^

There is a growing body of research demonstrating that simulated emergency scenarios are able to induce substantial acute stress levels in medical students, residents and attending physicians.^7^^-^^10^ Despite this evidence, the impact of acute stress on the health, performance and quality of care of physicians remains highly controversial.^11^^-^^13^ Regardless of the positive or negative effects of acute stress, the potential for simulated scenarios to provoke a stress response in learners has been suggested as an indicator of immersion, realism, engagement and/or fidelity.^14^^-^^20^

In acute care medicine, patient care almost always involves a multiprofessional team practice.^21^ Among the various professionals that participate in an emergency team, physicians and nurses have inspired an increasing number of studies related to their relationship and collaboration while working together.^22^^-^^24^ The responsibilities of doctors and nurses are inherently different, and these differences have led to negative stereotypes of each profession, disruptive communication and confusion regarding the roles that each team member should play.^25^^,^^26^ In fact, some studies

indicate that simulation-based training involving role-play strategies may improve confidence, attitudes and patient safety culture in multidisciplinary teams.^27^^-^^30^

To our knowledge, no previous research has investigated acute stress response in residents playing nurse roles in comparison to physician roles in simulated emergency scenarios. Assessing the acute stress levels evoked by different roles during simulations can provide relevant understanding regarding training immersion and engagement. The hypothesis of the present study is that acute stress levels induced in residents during an emergency simulation are equivalent between nurse and physician roles, indicating that both roles lead to similar engagement.

## Methods

### Study design and participants

This was a cross-sectional study carried out at the Simulation Center of University of São Paulo Medical School in São Paulo, Brazil. This study was approved by the Ethics and Research Committee of the University of São Paulo, and a written informed consent was obtained from all subjects. All second-year internal medicine residents rotating in an internal medicine ward were invited to voluntarily participate. Exclusion criteria were the following: pregnancy, infections of any kind, disease of the immune system, endocrine or metabolic diseases, use of any kind of medication (except for oral contraceptives) and inadequate salivary samples. The participation of the same resident in both simulated scenarios was allowed on different days. A total of 16 unique residents were enrolled in this study, and 8 of these participated in both scenarios. Therefore, a total of 24 observations (6 simulations) were analyzed. The median age of residents was 26.0 (IQR: 25.0-26.0) years, and 13 (54.2%) of them were female. At the time of the study enrollment, the participants had completed 15.5 (IQR: 15.0-16.0) months of residency. The emergency scenarios had a median duration of 25.0 (IQR: 23.0-27.0) minutes.

### Setting

In the simulation center, the scenarios were performed using a high-fidelity computer-based mannequin simulator, with the possibility of remote control of vital signs. All medications and equipment required during the scenarios were available, and the simulation room was set up similar to a real-life emergency room. The mannequin had a peripheral venous catheter already placed at the beginning of each scenario. Standardized physiologic responses to anticipated management steps were programmed into the scenario and activated by a physician facilitator. During the scenario, the facilitator remained in a one-way mirrored glass control room.

The scenarios used in this study were elaborated by three experienced simulation facilitators of the Clinical Emergency Discipline of University of São Paulo Medical School and were tested for two years prior to this study. The following emergency scenarios were applied:

         -  A fifty-five-year-old patient was admitted to the ER with precordial pain, with evidence of right ventricular myocardial infarction. During care, the patient develops cardiogenic shock and third-degree atrioventricular block, requiring volemic expansion, vasoactive drug and a transcutaneous cardiac pacing.

         -  A twenty-six-year-old patient was admitted to the ER with signs and symptoms of cocaine intoxication and develops unstable ventricular tachycardia. The patient presents with hypotension and acute pulmonary edema, requiring electrical cardioversion and invasive ventilation.

### Data collection methods

The researcher collected the following demographic variables after stress marker collection during T2: age, gender, time of residency and scenario duration. Acute stress levels were assessed using the following parameters:

**Heart rate:** This parameter was continuously measured using a heart rate monitor. The maximum between T1 and T2 was recorded from the watch system. Heart rate response was calculated as the percentage of variation from baseline to T2. For heart rate response, the maximum heart rate value was considered as T2. Average heart rate during overall simulation was also recorded.

**Systolic blood pressure (SBP):** This measure was obtained using an aneroid sphygmomanometer, and the mean of three measures was considered. Systolic blood pressure response was calculated as the percentage of variation from baseline to T2.

**Diastolic blood pressure (DBP):** This measure was obtained using an aneroid sphygmomanometer, and the mean of three measures was considered. Diastolic blood pressure response was calculated as the percentage of variation from baseline to T2.

**Salivary analyses:** Saliva samples were obtained by placing a specific swab under the tongue for 2 minutes. The samples were centrifuged at 1500 x g for 15 minutes at 4 °C and stored in a freezer at – 80 °C. The participants were required to abstain from eating, drinking (except water) and brushing teeth 1 hour before the material collection. Acute stress responses were calculated as the percentage of variation from baseline to T2, relating to the following markers:

   •   Salivary α-amylase (AA): measured using a kinetic colorimetric kit

   •   Salivary interleukin-1 β (IL-1β): measured using an immunoenzymatic kit

   •   Salivary cortisol (cortisol): measured using an immunoenzymatic kit

State component of State-Trait Anxiety Inventory: this widely used self-report inventory was applied to participants immediately after the end of the simulated scenario (T2), and its value score was considered as an acute stress indicator.

### Procedures

Each emergency team was formed by four residents, two playing the role of a nurse and two playing the role of a physician. The roles and scenarios were randomly chosen at the beginning of the simulation. The facilitator asked residents playing the nurse role to perform in the same way as nurses do in a real-life setting. The facilitator also stated that all decisions about task management should be defined by the team, without facilitator interference. Residents equally participated in both simulated scenarios (12 participants in each scenario). During simulated scenarios, all of the following procedures were performed exclusively by residents playing the physician role: orotracheal intubation, transcutaneous pacing and synchronized cardioversion. On the other hand, residents playing the nurse role performed exclusively the following procedures: medication preparation and infusion, infusion pump preparation and intubation material preparation. Residents playing the physician role took the leadership position in all simulations.

Residents were placed in a sitting position at rest for 5 minutes, and their baseline (T1) stress levels were measured between 1:30 pm and 2:00 pm. Before starting the scenario, all participants were oriented for 15 minutes by a physician facilitator about the simulation room setup, mannequin features and simulation methodology. Immediately after the end of the simulated emergency scenario (T2), stress levels were measured again. During the period between T1 and T2, participants remained with a heart rate monitor on. The end of the emergency care situation (T2) was considered when a) the mean arterial pressure was greater than 65 mmHg; b) oxygen saturation by pulse oximetry was greater than 90% after confirmed endotracheal tube position; c) there was return of spontaneous circulation maintained for at least 5 minutes or when terminated efforts and death was confirmed; or d) the patient was hemodynamically stable after therapeutic measures. Regardless of the clinical management, the scenario was finalized 30 minutes after its start.

### Statistical analysis

The sample size was calculated based on the nomogram proposed by Altman^31^and the results of a similar previous study.^32^ A total of 24 observations (12 in each group) were required to detect a difference of 15% in heart rate response between residents performing the nurse and physician roles.

The Shapiro-Wilk test was used to assess normality. Data were expressed as median and interquartile ranges (1st-3rd IQR) for continuous variables and as number and percentage for categorical variables. The Mann-Whitney U test was used to compare acute stress levels between the different roles. The U statistic value, difference between medians and 95% confidence interval (CI) for this difference were provided. Acute stress levels were defined as the percentage of variation from baseline (T1) to T2 regarding each parameter measuring stress. State component of State-Trait Anxiety Inventory was measured only during T2, and the average heart rate was measured during the entire period between T1 and T2. The level of statistical significance for this study was set at 0.05 (two-sided). All analyses were conducted using the SPSS Statistics software.

## Results

Comparing the group of residents playing the physician role with those playing the nurse role, we observed that the average heart rate during simulation was 101.5 (IQR: 92.0-104.0) versus 91.0 (IQR: 83.0-99.5) beats per minute, respectively, and this difference did not present statistical significance, diff: 10.5 (95%CI -2.0 to 23.0), U = 96.5, p = 0.16. Residents presented a State Anxiety Inventory score of 44.0 (IQR: 40.0-50.0) in the physician role versus 42.0 (IQR: 37.5-48.0) points in the nurse role, and this difference was not statistically significant, diff: 2.0 (95%CI -5.7 to 9.7), U = 89.5, p = 0.32.

**Figure 1:**
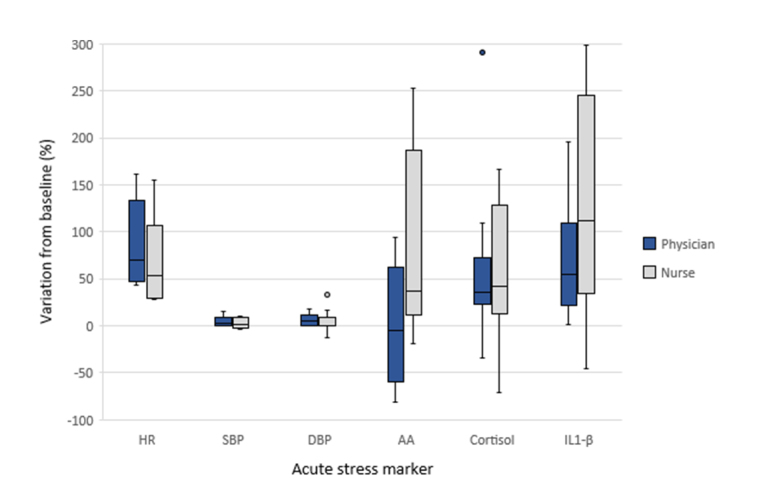
Variation of acute stress markers from baseline (T1) to T2 in both groups

In relation to the percent variation of the stress markers between baseline (T1) and T2, residents playing physician and nurse roles, respectively, presented the following: heart rate: 70.5% (IQR: 46.0-136.5) versus 53.0% (IQR: 29.5-117.0), diff: 17.50 (95%CI -50.9 to 85.9), U = 89.0, p=0.35; systolic blood pressure: 3.0% (IQR: 0.0-10.0) versus 2.0% (IQR: -2.0-9.0), diff: 1.0 (95%CI -6.8 to 8.8), U = 59.5, p=0.46; diastolic blood pressure: 5.5% (IQR: 0.0-13.5) versus 0.0% (IQR: 0.0-11.5), diff: 5.5 (95%CI -5.9 to 16.9), U = 91.5, p=0.27; cortisol: 35.3% (IQR: 22.2-83.5) versus 42.3% (IQR: 12.4-133.8), diff: -7.0 (95%CI -80.5 to 66.5), U = 64.0, p=0.08; α-amylase: -5.4% (IQR: -62.70-73.90) versus 37.5% (IQR: 11.0-186.8), diff: -42.9 (95%CI -186.3 to 100.6), U = 23.0, p=0.08; and interleukin-1 β: 54.4% (IQR: 21.9-109.3) versus 112.5% (IQR: 29.7-263.3), diff: -58.1 (95%CI -216.6 to 100.4), U = 24.0, p=0.27 (Figure 1).

## Discussion

The present study assessed acute stress levels in residents playing physician and nurse roles during two different simulated emergency scenarios. Since acute stress measurement presents an intricate complexity and there is no gold standard parameter to measure stress response,^33^ various markers involving cardiovascular, endocrine, immune and psychological parameters were used. The hypothesis was that both nurse and physician roles evoke similar acute stress in residents during emergency scenarios. The findings shown equivalent stress levels between both roles, indicating similar engagement.

A growing number of studies have reported that simulated scenarios may provoke substantial acute stress in doctors, nurses and other allied professionals.^7^^-^^10^^,^^34^^-^^36^ Some authors have suggested that the potential of a simulated scenario to induce acute stress in learners may be an indicator of realism, fidelity and engagement,^15^^,^^16^ especially in regards to what some authors have defined as psychological,^20^ phenomenal^19^ or high emotional and experiential fidelity.^17^

Despite this evidence, few studies have investigated stress levels in physicians playing different roles in simulated scenarios. Girzadas and colleagues,^32^ for instance, assessed acute stress levels in doctors while playing different roles (procedure chief, team leader or team member) during difficult airway scenarios. The authors observed that residents and medical students presented a significant increase in heart rate and self-reported stress level, but there was no correlation between the participant’s role and acute stress. In the present study, using a role-play strategy, it was observed that acute stress levels assessed by six different markers were not significantly different between residents playing nurse and physician roles. To our knowledge, this is the first study that assessed acute stress in doctors playing the nurse role.

In our experience using simulation in medical education, we have incorporated role-play and role reversal strategies for two different reasons. First, because the relationship between doctors and nurses in acute care settings has long been known to present many conflicts, misunderstandings and potential negative impact on quality of care.^22^^-^^26^ Additionally, some studies have suggested that simulation-based training involving role-play strategies may improve these interpersonal issues, as well as aspects related to patient safety.^27^^-^^30^^,^^37^ The immersion into the role of another professional allows doctors to gain familiarity with nurse perspectives,^38^^,^^39^ and this training may be very important for residents, who are still building their mental models related to working in multidisciplinary teams.

The second reason for using role-play is to enhance learner engagement in simulated scenarios. In fact, some studies have suggested that for teamwork training, the physical and environmental fidelities are less important than the psychological fidelity.^40^ Nikendei and colleagues reported that role-play activities provide an opportunity for improving the realism of training situations, while at the same time allowing learners to become more involved. The authors have added that, in an effort to create a more realistic training situation, role-playing proved to be a valuable learning tool.^38^ In accordance with these statements, the present research demonstrated that different roles may evoke similar acute stress in residents during emergency simulations, and it may indicate that both roles present similar simulation engagement.

The relationship between realism or fidelity and training effectiveness, as well as the factors that lead to a more realistic learning environment, are highly controversial.^40^^,^^41^ Likewise, the scientific literature has presented ambiguous findings regarding the impact of acute stress on performance and knowledge retention.^9^^,^^11^^,^^42^ Regardless of this wide range of controversial findings, the present study may contribute significantly to future research related to simulation and stress. Future studies may investigate, for instance, if similar stress levels found in both nurse and doctor roles are accompanied by similar performance and long-term knowledge retention. Research may also investigate if the learning related to the doctor-nurse relationship is transferred from simulation to real-life settings.

This study presents important limitations. First, the volunteer nature of inclusion may have favored the occurrence of selection bias. Second, since this study was carried out in a single center and included only second-year internal medicine residents, the findings may not necessarily generalize to other settings. In addition, the sample size of this study was relatively small. In future studies, larger samples may identify differences between groups that this research could not find.

## Conclusions

The findings of the present study suggest that different roles during emergency simulations evoke similar participants’ engagement, as indicated by acute stress levels. Role-play strategies can provide high psychological fidelity for simulation-based training, and these results reinforce the potential of role-play methodologies in medical education. Future studies can address the impact of acute stress on knowledge and skills retention as well as performance of residents.

### Acknowledgments

This research was funded by FAPESP (São Paulo Research Foundation).

### Conflict of Interest

The authors declare that they have no conflict of interest.

## References

[1] Huang GC, Sacks H, Devita M, Reynolds R, Gammon W, Saleh M, Gliva-McConvey G, Owens T, Anderson J, Stillsmoking K, Cantrell M, Passiment M (2012). Characteristics of simulation activities at North American medical schools and teaching hospitals: an AAMC-SSH-ASPE-AACN collaboration.. Simul Healthc.

[2] Khanduja PK, Bould MD, Naik VN, Hladkowicz E, Boet S (2015). The role of simulation in continuing medical education for acute care physicians: a systematic review.. Crit Care Med.

[3] Cook DA, Hatala R, Brydges R, Zendejas B, Szostek JH, Wang AT, Erwin PJ, Hamstra SJ (2011). Technology-enhanced simulation for health professions education: a systematic review and meta-analysis.. JAMA.

[4] McGaghie WC, Issenberg SB, Petrusa ER, Scalese RJ (2010). A critical review of simulation-based medical education research: 2003-2009.. Med Educ.

[5] Issenberg SB, McGaghie WC, Petrusa ER, Lee Gordon D, Scalese RJ (2005). Features and uses of high-fidelity medical simulations that lead to effective learning: a BEME systematic review.. Med Teach.

[6] McGaghie WC, Issenberg SB, Barsuk JH, Wayne DB (2014). A critical review of simulation-based mastery learning with translational outcomes.. Med Educ.

[7] Clarke S, Horeczko T, Cotton D, Bair A (2014). Heart rate, anxiety and performance of residents during a simulated critical clinical encounter: a pilot study.. BMC Med Educ.

[8] Keitel A, Ringleb M, Schwartges I, Weik U, Picker O, Stockhorst U, Deinzer R (2011). Endocrine and psychological stress responses in a simulated emergency situation.. Psychoneuroendocrinology.

[9] DeMaria S, Silverman ER, Lapidus KA, Williams CH, Spivack J, Levine A, Goldberg A (2016). The impact of simulated patient death on medical students' stress response and learning of ACLS.. Med Teach.

[10] Müller MP, Hänsel M, Fichtner A, Hardt F, Weber S, Kirschbaum C, Rüder S, Walcher F, Koch T, Eich C (2009). Excellence in performance and stress reduction during two different full scale simulator training courses: a pilot study.. Resuscitation.

[11] LeBlanc VR (2009). The effects of acute stress on performance: implications for health professions education.. Acad Med.

[12] Piquette D, Tarshis J, Sinuff T, Fowler RA, Pinto R, Leblanc VR (2014). Impact of acute stress on resident performance during simulated resuscitation episodes: a prospective randomized cross-over study.. Teach Learn Med.

[13] West CP, Tan AD, Habermann TM, Sloan JA, Shanafelt TD (2009). Association of resident fatigue and distress with perceived medical errors.. JAMA.

[14] Daglius Dias R, Scalabrini Neto A (2016). Stress levels during emergency care: A comparison between reality and simulated scenarios.. J Crit Care.

[15] Demaria S, Bryson EO, Mooney TJ, Silverstein JH, Reich DL, Bodian C, Levine AI (2010). Adding emotional stressors to training in simulated cardiopulmonary arrest enhances participant performance.. Med Educ.

[16] Bryson EO, Levine AI (2008). The simulation theater: a theoretical discussion of concepts and constructs that enhance learning.. J Crit Care.

[17] Rudolph JW, Simon R, Raemer DB (2007). Which reality matters? Questions on the path to high engagement in healthcare simulation.. Simul Healthc.

[18] Engstr?m H, Andersson Hagiwara M, Backlund P, Lebram M, Lundberg L, Johannesson M, Sterner A, Maurin S?derholm H (2016). The impact of contextualization on immersion in healthcare simulation.. Adv Simul.

[19] Dieckmann P, Gaba D, Rall M (2007). Deepening the theoretical foundations of patient simulation as social practice.. Simul Healthc.

[20] Rehmann A, Mitman R, Reynolds M. A handbook of flight simulation fidelity requirements for human factors research. Springfield, Virginia: DOT/FAA; 1995.

[21] Malt G (2015). Cochrane review brief: interprofessional education: effects on professional practice and healthcare outcomes.. Online J Issues Nurs.

[22] Tang CJ, Chan SW, Zhou WT, Liaw SY (2013). Collaboration between hospital physicians and nurses: an integrated literature review.. Int Nurs Rev.

[23] Adler-Milstein J, Neal K, Howell MD (2011). Residents' and nurses' perceptions of team function in the medical intensive care unit.. J Crit Care.

[24] Weller JM, Janssen AL, Merry AF, Robinson B (2008). Interdisciplinary team interactions: a qualitative study of perceptions of team function in simulated anaesthesia crises.. Med Educ.

[25] McKay K, Narasimhan S. Bridging the gap between doctors and nurses. J Nurs Educ Pract. 2012;2(4):52-55.

[26] Muller-Juge V, Cullati S, Blondon KS, Hudelson P, Maître F, Vu NV, Savoldelli GL, Nendaz MR (2014). Interprofessional collaboration between residents and nurses in general internal medicine: a qualitative study on behaviours enhancing teamwork quality.. PLoS ONE.

[27] Mason VM, Lyons P (2013). Use of simulation to practice multidisciplinary anaphylaxis management.. Dimens Crit Care Nurs.

[28] Featherstone P, Smith GB, Linnell M, Easton S, Osgood VM (2005). Impact of a one-day inter-professional course (ALERT) on attitudes and confidence in managing critically ill adult patients.. Resuscitation.

[29] Reeves S, Perrier L, Goldman J, Freeth D, Zwarenstein M (2013). Interprofessional education: effects on professional practice and healthcare outcomes (update).. Cochrane Database Syst Rev.

[30] Murdoch NL, Bottorff JL, McCullough D (2014). Simulation education approaches to enhance collaborative healthcare: a best practices review.. Int J Nurs Educ Scholarsh.

[31] Altman DG. Practical statistics for medical research. London, UK: Chapman & Hall; 1991.

[32] Girzadas DV, Delis S, Bose S, Hall J, Rzechula K, Kulstad EB (2009). Measures of stress and learning seem to be equally affected among all roles in a simulation scenario.. Simul Healthc.

[33] Goldstein DS, Kopin IJ (2007). Evolution of concepts of stress.. Stress.

[34] McKay KA, Buen JE, Bohan KJ, Maye JP (2010). Determining the relationship of acute stress, anxiety, and salivary alpha-amylase level with performance of student nurse anesthetists during human-based anesthesia simulator training.. AANA J.

[35] Judd BK, Alison JA, Waters D, Gordon CJ (2016). Comparison of psychophysiological stress in physiotherapy students undertaking simulation and hospital-based clinical education.. Simul Healthc.

[36] Bong CL, Lightdale JR, Fredette ME, Weinstock P (2010). Effects of simulation versus traditional tutorial-based training on physiologic stress levels among clinicians: a pilot study.. Simul Healthc.

[37] Wong AH, Gang M, Szyld D, Mahoney H (2016). Making an "attitude adjustment": using a simulation-enhanced interprofessional education strategy to improve attitudes toward teamwork and communication.. Simul Healthc.

[38] Nikendei C, Zeuch A, Dieckmann P, Roth C, Schäfer S, Völkl M, Schellberg D, Herzog W, Jünger J (2005). Role-playing for more realistic technical skills training.. Med Teach.

[39] Baile WF, Blatner A (2014). Teaching communication skills: using action methods to enhance role-play in problem-based learning.. Simul Healthc.

[40] Beaubien JM, Baker DP (2004). The use of simulation for training teamwork skills in health care: how low can you go?. Qual Saf Health Care.

[41] Rystedt H, Sjöblom B (2012). Realism, authenticity, and learning in healthcare simulations: rules of relevance and irrelevance as interactive achievements.. Instr Sci.

[42] Joëls M, Pu Z, Wiegert O, Oitzl MS, Krugers HJ (2006). Learning under stress: how does it work?. Trends Cogn Sci (Regul Ed ).

